# How effective are APRI, FIB-4, FIB-5 scores in predicting liver fibrosis in chronic hepatitis B patients?

**DOI:** 10.1097/MD.0000000000030488

**Published:** 2022-09-09

**Authors:** Ferdane Pirincci Sapmaz, Galip Büyükturan, Yusuf Serdar Sakin, İsmail Hakki Kalkan, Pinar Atasoy

**Affiliations:** a Gastroenterology Department, Gülhane Education and Training Hospital, Ankara, Turkey; b Gastroenterology Department, TOBB University of Economics and Technology, Ankara, Turkey; c Pathology Department, Kirikkale University Faculty of Medicine, Kirikkale, Turkey.

**Keywords:** APRI, chronic hepatitis B, FIB-4, FIB-5

## Abstract

Liver fibrosis is the most important factor in the prognosis and treatment plan of patients with chronic hepatitis B (CHB). Aspartate aminotransferase (AST)-to-platelet ratio index (APRI), fibrosis index based on 4 factors (FIB-4), and fibrosis index based on 5 factors (FIB-5) scores are noninvasive fibrosis markers, and previous comparative studies have shown that they are as effective as liver biopsy in detecting liver fibrosis in different liver diseases. The aim of our study is to investigate whether existing scoring systems are effective in demonstrating fibrosis in CHB patients and to compare the APRI, FIB 4, and FIB 5 scores in differentiating early and advanced fibrosis in 123 patients who underwent liver biopsy for CHB infection. APRI, FIB-4, and FIB-5 scores of patients who underwent liver biopsy due to CHB were calculated by means of calculators and recorded to be compared with liver biopsies in terms of fibrosis scoring. One hundred twenty-three patients who underwent liver biopsy due to chronic hepatitis B were included in the study. APRI (area under the receiver-operating characteristic [ROC] curve 0.728), FIB-4 (area under the ROC curve 0.693) and FIB-5 (area under the ROC curve 0.643) scores were evaluated as significant predictors of advanced fibrosis. The scoring system with the highest positive and negative predictive value was evaluated as FIB-4. APRI, FIB-4, and FIB-5 scoring systems are appropriate scoring systems in the assessment of advanced fibrosis in patients with CHB. Our study is the first to compare APRI, FIB-4, and FIB-5 values in CHB patients, and more comprehensive studies are needed.

## 1. Introduction

Approximately, 350 to 400 million people in the world population are known to be chronic HBV surface antigen carriers.^[1]^ Also chronic hepatitis B (CHB) is one of the most common causes of liver fibrosis and cirrhosis. In patients with chronic liver disease, histological evaluation of the liver is of essential importance in order to reveal the degree of fibrosis. In particular, in liver disease due to hepatitis B, initiation of treatment, referral, and patient follow-up are determined by the degree of fibrosis of the liver. Percutaneous liver biopsy has been the gold standard for assessment of fibrosis; however, limitations of this procedure include cost, risk of serious complications, sampling errors and inter- and intra-observer variations.^[2,3]^ Due to the current handicaps of liver biopsy, noninvasive scoring systems based on laboratory tests have been defined in the evaluation of liver fibrosis.

Two commonly used scoring systems for chronic liver diseases are the “aspartate aminotransferase (AST)-to-platelet ratio index” (APRI), the “fibrosis index based on 4 factors” (FIB-4).^[4]^ APRI and FIB-4 scores are the most commonly used scoring systems to predict liver fibrosis.^[5]^ The “fibrosis index based on 5 factors” (FIB-5) is a scoring system that has just started to be used and needs more study in patients with CHB.^[6]^

However, APRI, FIB-4, and FIB-5 scoring systems seem to be insufficient in some studies to differentiate early stage fibrosis from advanced fibrosis due to their low sensitivity and positive predictive values.^[7]^

Our study was designed to evaluate how effective the APRI, FIB-4, and FIB-5 scoring systems are in predicting liver fibrosis in CHB patients who underwent liver biopsy for treatment decision.

## 2. Material and methods

### 2.1. Ethical approval

This cross-sectional study included consecutive adult patients with CHB who had undergone percutaneous liver biopsy at Kirikkale University of Medicine Gastroenterology Department from September 2012 to October 2014. This study was approved by the Kirikkale University of Medicine Local Ethics Committee (no: KADB-F.03-R.03/ 04.07.2014). Informed consent was not obtained as it was a retrospective study.

### 2.2. Patients and data

A total of 123 patients were included in the study. Demographic data and laboratory parameters of all patients were documented. Laboratory data included liver function tests, complete blood count, international normalized ratio, albumin and HBV DNA levels. All patients included in the study, who were evaluated as CHB infection, were HBV surface antigen positive for more than 6 months and HBV DNA levels could be detected by real-time polymerase chain reaction.

### 2.3. Scoring systems

The following non-invasive markers of fibrosis were examined;

**APRI Scores**: (AST [IU/L]/upper normal limit of AST [IU/L])/platelets [10^3^/mm^3^]

**FIB 4 Scores**: (age [years] × AST [IU/L])/(platelets [10^3^/mm^3^] × ALT (alanine aminotransferase) ([IU/L])

**FIB-5 Scores**: albumin (g/L) × 0.3 + platelet count (10^9^/L) × 0.05 − alkaline phosphatase (IU/L) × 0.014 + AST/ALT ratio × 6 + 14

### 2.4. Biopsies

Liver biopsy specimens were analyzed by an experienced pathologist, who was blinded to the clinical results. Liver fibrosis and necroinflammatory activity were evaluated with the METAVIR classification.^[7]^ Fibrosis scores were divided into 2 groups as F0-1 (non-significant fibrosis [NSF]) and F2-4 (significant fibrosis [SF]).

### 2.5. Statistical analysis

The data of the research were analyzed with the help of SPSS Statistics v26.0 (IBM SPSS Statistics for Windows, Version 26.0. Armonk, NY) package program. The significance level of .05 was taken as a basis in the analysis of the data. In cases where the significance of the difference between the means of 2 separate groups was investigated, the Independent Samples *t* test was used, and before the use of this test, the normality test was carried out with the help of Kolmogorov–Smirnov and Shapiro–Wilk tests. When comparing the distribution of patients with mild and severe fibrosis in terms of gender, a crosstab was drawn. The “chi-square test” was used because the data could meet the assumptions of this test. In order to reveal the performance of the diagnostic tests, to determine the threshold value and to reveal numerical results regarding the specificity-sensitivity of the test, receiver-operating characteristic (ROC) analysis was performed and the ROC curve was drawn with this analysis. The area under the ROC curve was interpreted as the accuracy rate in separating individuals with mild and severe fibrosis.

## 3. Results

### 3.1. Patient characteristics

Data of 123 patients were included in the study and analyzed. In the histopathological evaluation, there was 1 patient with F0 (0.8%), 60 patients with F1 (48.8%), 32 patients with F2 (26%), 19 patients with F3 (15.4%), 8 patients with F4 (6.5%), and 3 patients with F5 (2.4%) according to the fibrosis stage (Table 1).

**Table 1:** Frequency distribution of all studied patients according to Ishak fibrosis score.

		Frequency	Percent	Valid percent	Cumulative percent
Valid	0	1	0.8	0.8	0.8
	1	60	48.8	48.8	49.6
	2	32	26.0	26.0	75.6
	3	19	15.4	15.4	91.1
	4	8	6.5	6.5	97.6
	5	3	2.4	2.4	100.0
	6	0	0.0	0.0	100.0
	Total	123	100.0	100.0	

Group 1 = non-significant fibrosis (Ishak fibrosis score: 0–2), Group 2 = significant fibrosis (Ishak fibrosis score: 3–6).

### 3.2. Biochemical parameters

AST (49.74 ± 51.8 IU/mL vs 97.57 ± 103.91 IU/mL, respectively; *P* = .021), ALT (69.92 ± 85.70 IU/mL vs 155.47 ± 163.44 IU/mL, respectively; *P* = .010), alkaline phosphatase (82.48 ± 37.44 IU/L vs 102.70 ± 58.92 IU/L, respectively; *P* = .029), gamma glutamyl transferase (36.89 ± 43.24 IU/L vs 60.37 ± 43.37 IU/L, respectively; *P* = .011), alpha fetoprotein (2.52 ± 1.32 ng/mL vs 3.57 ± 1.78 ng/mL, respectively; *P* = .001) were significantly higher in SF group as compared with NSF group. Platelet count was significantly lower in SF group (231.70 ± 71.02 vs 194 ± 78.20 (×10^9^/L) respectively; *P* = .015).

The demographic profile and laboratory data of patients is shown in Table 2.

**Table 2:** Baseline characteristics of chronic hepatitis B patients with hepatic fibrosis regards 2 main classifications.

Parameters	Non-significant fibrosis group (n = 93)	Significant fibrosis group (n = 30)	*P* value
Gender (M/F)	50/43	20/10	.215
Age (yr)	47.35 (±10.01)	47.23 (±8.85)	.953
BMI (kg/m²)	26.51 (±3.81)	27.85 (±3.99)	.099
Blood glucose (mg/dL)	94.68 (±22.32)	95.77 (±15.43)	.804
Urea (mg/dL)	31.18 (±8.47)	30.37 (±8.85)	.0651
Creatinine (mg/dL)	0.78 (±0.19)	0.78 (±0.21)	.996
AST (U/L)	49.74 (±51.58)	97.57 (±103.91)	.021*
ALT (U/L)	69.92 (±85.70)	155.47 (±163.44)	.010*
ALP (U/L)	82.48 (±37.44)	102.70 (±58.92)	.029*
GGT (U/L)	36.89 (±43.24)	60.37 (±43.37)	.011*
Total bilirubin (mg/dL)	0.98 (±1.64)	0.81 (±0.40)	.585
Albumin (g/L)	4.4 (±0.44)	4.2 (±0.38)	.090
INR	1.02 (±0.15)	1.07 (±0.15)	.162
PLT (×10³/mm³)	231.70 (±71.02)	194 (±78.20)	.015*
Leukocyte (/mm³)	6755.91 (±2133.35)	6986.00 (±1718.08)	.592
Sedimentation (mm/h)	9.6 (±6.77)	10.13 (±7.20)	.714
AFP (ng/mL)	2.52 (±1.32)	3.57 (±1.78)	.001*
CRP (mg/L)	5.11 (±3.59)	5.13 (±2.49)	.984
HBV-DNA	217,455 (±590,801)	332,938 (±349,745)	.313
APRI	0.65 (±0.83)	1.34 (±1.32)	.010*
FIB4	1.36 (±0.87	1.98 (±1.12)	.020*
FIB5	4.37 (±4.82)	2.68 (±4.21)	.088

AFP = alpha fetoprotein, ALP = alkaline phosphatase, ALT = alanine aminotransferase, APRI = aspartate aminotransferase-to-platelet ratio index, AST = aspartate aminotransferase, BMI = body mass index, CRP = C reactive protein, FIB-4 = fibrosis index based on 4 factors, FIB-5 = fibrosis index based on 5 factors, GGT = gamma glutamyl transferase, HBV-DNA= hepatitis B virus DNA, INR = international normalized ratio, PLT = platelets.

*means: *P* < 0.05.

APRI (0.65 ± 0.83 vs 1.34 ± 1.32, respectively; *P* = .010) and FIB 4 (1.36 ± 0.87 vs 1.98 ± 1.12, respectively; *P* = .020) levels were found to be significantly higher in the SF group than in the NSF group. Although FIB 5 (4.37 ± 4.82 vs 2.68 ± 4.21, respectively; *P* = .088) levels were found to be lower in the SF group than in the NSF group, no significant relationship could be found.

**Figure 1.**
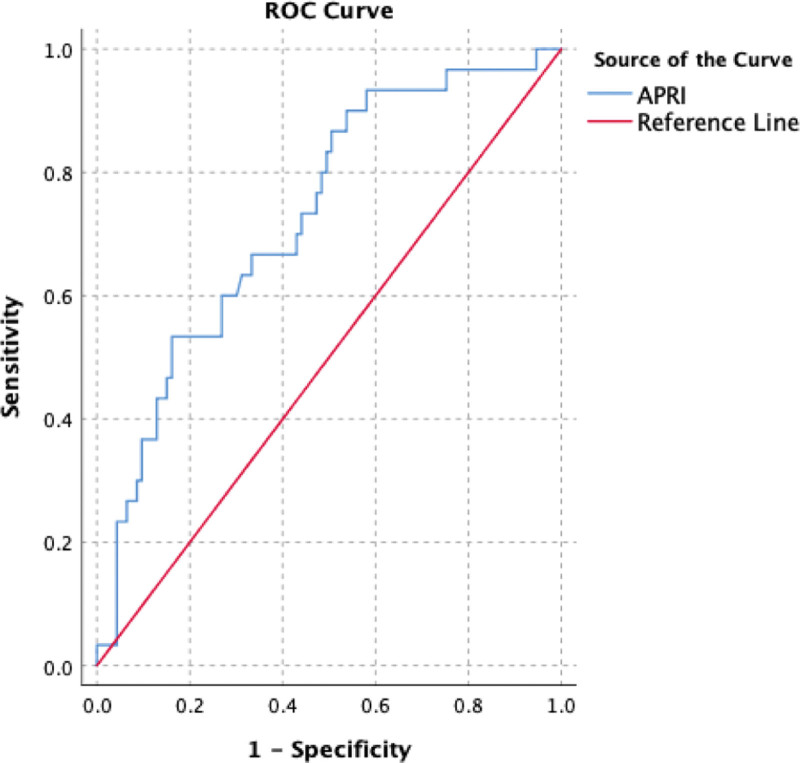
ROC curve generated by APRI for differentiation between significant and non-significant fibrosis. APRI = aspartate aminotransferase-to-platelet ratio index, ROC = receiver-operating characteristic.

**Figure 2.**
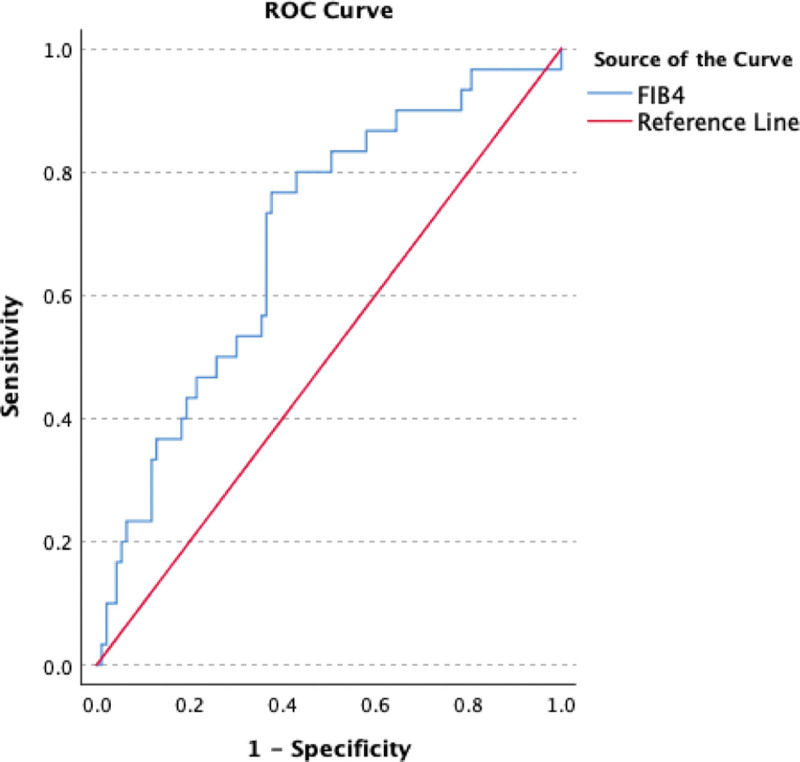
ROC curve generated by FIB-4 for differentiation between significant and non-significant fibrosis. FIB-4 = fibrosis index based on 4 factors, ROC = receiver-operating characteristic.

**Figure 3.**
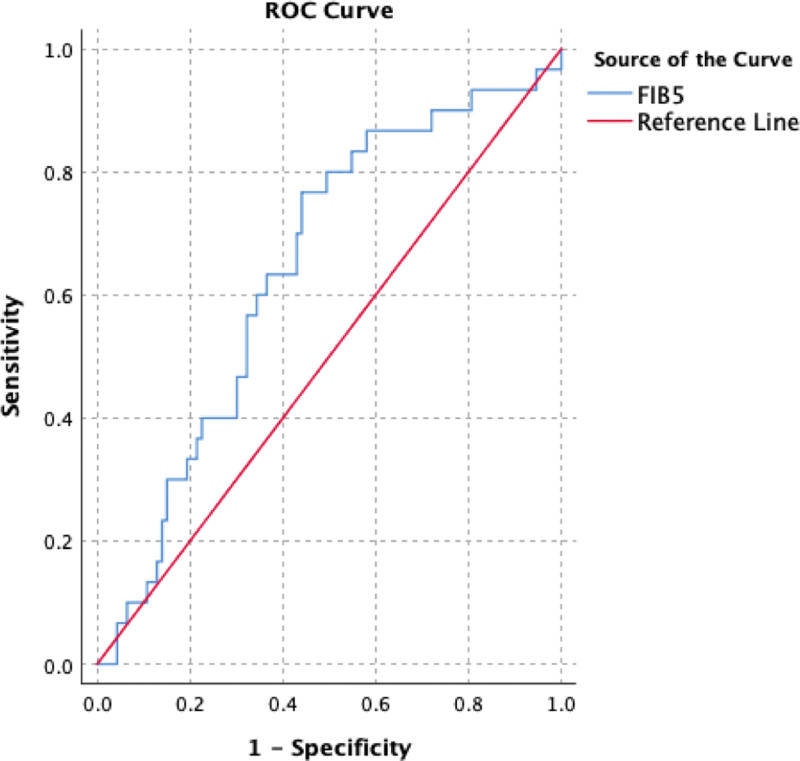
ROC curve generated by FIB-5 for differentiation between significant and non-significant fibrosis. FIB-5 = fibrosis index based on 5 factors, ROC = receiver-operating characteristic.

The ROCs of APRI, FIB-4, and FIB-5 are as shown in Figures 1–3 (Table 3). APRI had sensitivity, specificity, positive predictive value (PPV), negative predictive value (NPV), and AUROC of 73.3%, 55.9%, 34.9%, 86.6%, and 0.72, respectively. FIB-4 had sensitivity, specificity, PPV, NPV, and AUROC of 80%, 57%, 37.5%, 89.8%, and 0.69, respectively. While FIB-5 had sensitivity, specificity, PPV, NPV, and AUROC of 83.3%, 45.2%, 32.8%, 89.4%, and 0.64, respectively.

**Table 3:** Performance characteristics of both APRI, FIB-4, and FIB-5 to differentiate between F0, F1 and other fibrosis stages.

Test	*P*	Cut-off	95% CI	AUC	St. error	Sensitivity (%)	Specificity (%)	PPV (%)	NPV (%)
APRI	.010	0.424	0.627–0.829	0.728	0.052	73.3	55.9	34.9	86.6
FIB-4	.020	1.22	0.586–0.800	0.693	0.054	80	57	37.5	89.8
FIB-5	.088	5.076	0.533–0.752	0.643	0.056	83.3	45,2	32.8	89.4

AUC = area under the ROC curve, APRI = aspartate aminotransferase-to-platelet ratio index, CI = confidence interval, FIB-4 = fibrosis index based on 4 factors, FIB-5 = fibrosis index based on 5 factors, NPV = negative predictive value, PPV = positive predictive value.

## 4. Discussion

CHB infection remains among the most common causes of cirrhosis worldwide. Therefore, early and noninvasive diagnosis of fibrosis in CHB infection is of great importance for rapid initiation of treatment. On the other hand, it seems possible that noninvasive tests used in clinical practice will lead to further evaluation of these patients by creating overdiagnosis. However, when these noninvasive tests are compared with liver biopsy, they seem to be beneficial in terms of preventing complications that may develop due to liver biopsy, as well as providing short-term cost savings. Therefore, it is of great importance to optimize the cut-off values of APRI, FIB 4, and FIB 5 scores to reach the closest values that correlate with liver biopsy findings.

The aim of this study was to compare the APRI, FIB 4, and FIB 5 scores in differentiating early and advanced fibrosis in 123 patients who underwent liver biopsy for CHB infection.

We found a high correlation between advanced fibrosis and patients’ ALT, AST, gamma glutamyl transferase, alkaline phosphatase, alpha fetoprotein levels, platelets count, APRI, FIB 4 scores in our study. As expected, platelets count of hepatitis B patients with advanced fibrosis were lower as compared with low fibrosis stages.

In our study, it was revealed that the APRI score is a significant indicator in demonstrating advanced stage fibrosis in patients with CHB with AUROC of 0.728. Similarly, in a meta-analysis,^[8]^ which included 9 studies, the APRI gave AUROCs for SF and cirrhosis of 0.79 and 0.75, respectively. In the same meta-analysis, for SF, an APRI cut-off of 0.5 had a sensitivity of 84% and a specificity of 41%, while a cut-off of 1.5 had a sensitivity of 49% and a specificity of 84%.

In our study sensitivity and specificity of APRI score in predicting SF in CHB patients were 73.3% and 55.9%, respectively, for an APRI score cut-off of 0.4.

In another meta-analysis,^[9]^ sensitivity and specificity values were 70% and 60%, 50% and 83%, and 36.9% and 92.5% for fibrosis, advanced fibrosis, and cirrhosis, in the ranges of 0.5, 1, and 1.5 APRI thresholds has been reported, respectively.

In addition, in a meta-analysis conducted with the sensitivity of noninvasive scoring systems in the detection of esophageal varices, which has a very important place in the definition of liver cirrhosis, APRI, and FIB-4 scores had low to moderate diagnostic accuracy in predicting the presence of varices in liver cirrhosis.^[10]^

Our study showed that FIB-4 scores are also a significant parameter in demonstrating advanced fibrosis in CHB with AUROC of 0.693. In our study sensitivity and specificity of FIB-4 score in predicting SF in CHB patients were 80% and 57%, respectively, for an FIB-4 score cut-off of 1.2.

In another retrospective study^[11]^ conducted with 138 liver-biopsied CHB patients, it was shown that the FIB-4 index could classify patients with moderate fibrosis with an AUROC value of 0.81. Also a cut-off value ≤1.45 could differentiate moderate from severe fibrosis with a sensitivity of 71% and a specificity of 73%.

In a meta-analysis conducted in 2014, an AUROC value of 0.78 was found in SF for cut-off values in the range of 1.45 to 1.62, while a cut-off value of 2.9 to 3.6 for cirrhosis was found to be 0.89.^[12]^

The AUROC of FIB-5 for differentiating NSF from SF was 0.64. FIB-5 values at a cutoff level of 5.07 showed sensitivity 83.3% with a specificity of 45.2% for the differentiation between significant and NSF.,

In a study on FIB-5 scores in CHB patients, when the cut-off value was determined as 7.05, the specificity was found to be 98.8% and the sensitivity 23.1% in distinguishing between significant and in SF.^[13]^

The distinction between FIB-5 and FIB-4 scores is the presence of serum albumin and alkaline phosphatase levels in the fib-5 score, which indicates hepatic synthesis function.

The FIB-5 score, like other scoring systems, is a noninvasive, easy, and inexpensive method in clinical practice. However, since there are not enough data on other liver diseases, especially in CHB, studies with much larger patient groups are needed.

### 4.1. Study limitations

Our study has several limitations. One of them was the low number of our cases. More meaningful studies can be done in larger case series.

In addition, there are no world-accepted cut-off values for the tests we work with. Therefore, we designed our study according to the cut-off levels with the most appropriate sensitivity and specificity.

## 5. Conclusion

In summary, APRI, FIB-4, and FIB-5 scoring systems are available for the determination of fibrosis in CHB patients. In our study, FIB-5 was also found to be a useful scoring system in detecting and staging fibrosis; however, there are not enough studies on FIB-5 levels in the literature. More comprehensive studies on this subject are needed.

## Main points

1. The use of non-invasive tests in the evaluation of the fibrosis score that guides the treatment in CHB patients is very important to prevent liver biopsy complications.
2. APRI, FIB-4, FIB-5 scores are very effective tests at various cut-off values in demonstrating fibrosis. Our study is the first to compare these tests in these CHB patients.
3. Although there are many tests showing fibrosis, there is still no clear information about which cut-off level they should be evaluated.
4. There is a need for detailed meta-analyses regarding optimal cut-off values in these tests.

## Author contributions

İHK and FS: concept; İHK and FS: design; İHK, FS, and PA: materials; İHK and FS: data collection and/or processing; FS and YSS: analysis and/or interpretation; YSS and GB: literature search; FS: writing manuscript; İHK, FS, and GB: critical review.

**Conceptualization:** Ferdane Pirincci Sapmaz, Galip Büyükturan, Yusuf Serdar Sakin, İsmail Hakki Kalkan, Pinar Atasoy.

**Data curation:** Ferdane Pirincci Sapmaz.

**Formal analysis:** Galip Büyükturan.

**Methodology:** Yusuf Serdar Sakin.

**Project administration:** Pinar Atasoy.

**Software:** İsmail Hakki Kalkan.

**Supervision:** Yusuf Serdar Sakin, İsmail Hakki Kalkan, Pinar Atasoy.

Visualization: Ferdane Pirincci Sapmaz, İsmail Hakki Kalkan.

Writing – original draft: Ferdane Pirincci Sapmaz.

Writing – review & editing: Ferdane Pirincci Sapmaz.

## References

[1] McMahon BJ. The natural history of chronic hepatitis B virus infection. Hepatology. 2009;49:S45–55. 19399792 10.1002/hep.22898

[2] Cadranel JF Rufat P Degos F. Practices of liver biopsy in France: results of a prospective nationwide survey. For the Group of Epidemiology of the French Association for the Study of the Liver (AFEF). Hepatology. 2000;32:477–81. 10960438 10.1053/jhep.2000.16602

[3] French METAVIR Cooperative Study Group. Intraobserver and interobserver variations in liver biopsy interpretation in patients with chronic hepatitis C. The French METAVIR cooperative study group. Hepatology. 1994;20:15–20. 8020885

[4] European Association for Study of Liver. Asociación Latinoamericana para el Estudio del Higado. EASL-ALEH clinical practice guidelines: non-invasive tests for evaluation of liver disease severity and prognosis. J Hepatol. 2015;63:237–64. 25911335 10.1016/j.jhep.2015.04.006

[5] Erdogan S Dogan HO Sezer S. The diagnostic value of non-invasive tests for the evaluation of liver fibrosis in chronic hepatitis B patients. Scand J Clin Lab Invest. 2013;73:300–8. 23514016 10.3109/00365513.2013.773592

[6] Attallah AM Shiha GE Omran MM. A discriminant score based on routine laboratory blood tests for accurate diagnosis of severe fibrosis and/or liver cirrhosis in Egyptian patients with chronic hepatitis C. Hepatol Res. 2006;34:163–9. 16478676 10.1016/j.hepres.2005.12.004

[7] Amorim TG Staub GI Lazzarotto C. Validation and comparison of simple noninvasive models for the prediction of liver fibrosis in chronic hepatitis C. Ann Hepatol. 2012;11:855–61. 23109448

[8] Jin W Lin Z Xin Y. Diagnostic accuracy of the aspartate aminotransferase-to-platelet ratio index for the prediction of hepatitis B-related fibrosis: a leading meta-analysis. BMC Gastroenterol. 2012;12:14. 22333407 10.1186/1471-230X-12-14 PMC3306191

[9] Xiao G Yang J Yan L. Comparison of diagnostic accuracy of aspartate aminotransferase to platelet ratio index and fibrosis-4 index for detecting liver fibrosis in adult patients with chronic hepatitis B virus infection: a systemic review and meta-analysis. Hepatology. 2015;61:292–302. 25132233 10.1002/hep.27382

[10] Deng H Qi X Guo X. Diagnostic accuracy of APRI, AAR, FIB-4, FI, King, Lok, Forns, and FibroIndex scores in predicting the presence of esophageal varices in liver cirrhosis: a systematic review and meta-analysis. Medicine (Baltim). 2015;94:e1795. 10.1097/MD.0000000000001795 PMC4620760 26496312

[11] Mallet V Dhalluin-Venier V Roussin C. The accuracy of the FIB-4 index for the diagnosis of mild fibrosis in chronic hepatitis B. Aliment Pharmacol Ther. 2009;29:409–15. 19035983 10.1111/j.1365-2036.2008.03895.x

[12] Li Y Chen Y Zhao Y. The diagnostic value of the FIB-4 index for staging hepatitis B-related fibrosis: a meta-analysis. PLoS One. 2014;9:e105728. 25165830 10.1371/journal.pone.0105728 PMC4148327

[13] Metwally K Elsabaawy M Abdel-Samiee M. FIB-5 versus FIB-4 index for assessment of hepatic fibrosis in chronic hepatitis B affected patients. Clin Exp Hepatol. 2020;6:335–8. 33511281 10.5114/ceh.2020.102157 PMC7816634

